# Evaluation of Apical Leakage in Root Canals Obturated with Three Different Sealers in Presence or Absence of Smear Layer

**Published:** 2015-03-18

**Authors:** Hadi Mokhtari, Shahriar Shahi, Maryam Janani, Mohammad Frough Reyhani, Hamid Reza Mokhtari Zonouzi, Saeed Rahimi, Hamid Reza Sadr Kheradmand

**Affiliations:** a *Dental and Periodontal Research Center, Department of Endodontics, Faculty of Dentistry, Tabriz University of Medical Sciences, Tabriz, Iran; *

**Keywords:** Apical Microleakage, Gutta-Percha, Root canal Filling, Root Canal Sealer, Smear Layer

## Abstract

**Introduction:** Microleakage can result in failure of endodontic treatment. An important characteristic of endodontic sealer is sealing ability. The aim of this experimental study was to compare the apical leakage of teeth obturated with gutta-percha and three different sealers (resin- and zinc oxide eugenol-based) with/without smear layer (SL). **Materials and Methods:** In this study, 100 single-rooted teeth were used after cutting off their crowns. Cleaning and shaping was carried out with step-back technique and the samples were randomly divided into three groups (*n*=30) which were then divided into two subgroups (*n*=15) according to the presence/absence of SL. Two negative and positive control groups (*n*=5) were also prepared. In the various groups, the canals were obturated with gutta-percha and either of the test sealers (AH-26, Adseal or Endofill). The samples were submerged in India ink for 72 h. Then they were longitudinally sectioned and observed under a stereomicroscope at 20× magnification. Data were analyzed with descriptive statistical methods and one-way ANOVA. The significance level was set at 0.05. **Results:** The mean penetration length of dye in AH-26, Adseal and Endofill samples were 2.53, 2.76 and 3.03 mm, respectively. The differences between three groups were not significant (*P*>0.05); also, the mean dye penetration in AH-26, Adseal and Endofill samples in presence or absence of the SL was not significantly different. **Conclusion:** AH-26, Adseal and Endofill were similarly effective in prevention of apical microleakage. Differences in the mean dye penetration between the groups with/without the SL were not statistically significant.

## Introduction

Complete obturation and hermetic seal of the root canal system is a major goal in root canal treatment [1]. Microleakage in the root canal is a complex subject that can be affected by many variables such as root filling techniques and the physical and chemical properties of used sealers [2-4].

Several techniques have been developed to improve the seal of the prepared root canals [1]. The most common obturation technique is the cold lateral compaction of gutta-percha in combination with an insoluble root canal sealer [5]. The sealer is very important for long-term seal of the root canal filling because it adheres gutta-percha to the root canal dentin and fills irregularities and spaces among gutta-percha cones and between the root canal walls and fillings [4, 6]. 

Zinc oxide eugenol (ZOE)-based sealers were introduced by Grossman in 1936, to be used in conjunction with the gutta-percha or silver cones. Endofill is a commonly used ZOE-based sealer, available in a powder-liquid form [7]. Root canal sealers based on epoxy-resin are employed because of their good physicochemical properties and adhesion [8, 9]. AH-26 is one of the most common epoxy-resin sealers, which is claimed to provide excellent sealing properties [10, 11]. Adseal is also another resin-based sealer that contains bismuth phosphate and zinc-oxide mixed with vinyl polymer available in two paste-containing tubes [12].

The advantages and disadvantages of removing the smear layer (SL) in instrumented root canals is still a controversial issue [13, 14]. It is stated that removal of the SL significantly increases the push-out bond strength of root filling materials to root dentine [15] and its presence contributes to leakage and compromises the seal of the root canal filling [16]. However, conflicting findings in this regard might have resulted from the use of different forms of chelating agents to remove the SL, the type of used sealer (the resin-based sealers need to penetrate the patent dentinal tubules), the film thickness and flow rate of the sealer and the root filling technique [3, 5, 13, 17]. 

The aim of this experimental study was to compare the apical leakage of root canals obturated with gutta-percha and three different root canal sealers including AH-26, Adseal or Endofill in the presence/absence of the SL, using dye penetration method.

## Materials and Methods

In the present study, a total of 100 extracted single-rooted anterior teeth with fully developed apices were used. The teeth were cleaned in normal saline solution and disinfected in 5.25% NaOCl. The crowns were removed at cemento-enamel junction (CEJ) by a fissure diamond bur under water spray; apical patency was established with a #10 K-file (Dentsply-Maillefer, Ballaigues, Switzerland) and the length of each canal was determined by placing a #15 K-file into the canal until it became visible at the apical foramen. The working length (WL) was determined 1 mm shorter than this length. Gates-Glidden burs #2 to 4 (Maillefer, Ballaigues, Switzerland) were used to flare the coronal third of the canals. Cleaning and shaping was done with step-back technique and the master apical file (MAF) was kept at #40. During instrumentation 5 mL of 5.25% NaOCl solution was used to irrigate the canals.

**Table 1:** Mean (SD) of dye penetration (mm) in three groups (SL+=with smear layer and SL-=without smear layer)

**Group (N)**	**Mean (SD)**
**AH-26/SL** ^+^ ** (15)**	2.66 (0.89)
**AH-26/SL** ^-^ ** (15)**	2.40 (0.91)
**Total (30)**	2.53 (0.89)
**Adseal/SL** ^+^ ** (15)**	2.73 (0.88)
**Adseal/SL** ^-^ ** (15)**	2.80 (1.01)
**Total (30)**	2.76 (0.93)
**Endofill/SL** ^+^ ** (15)**	3.06 (0.79)
**Endofill/SL** ^-^ ** (15)**	3.00 (0.75)
**Total (30)**	3.03 (0.76)
**SL** ^+^ ** (45)**	2.82 (0.86)
**SL** ^-^ ** (45)**	2.73 (0.91)
**Total (90)**	2.77 (0.88)

The teeth were then randomly divided into three experimental groups (*n*=30) and two negative and positive control groups (*n*=5). Then each of the experimental groups were divided into two subgroups (*n*=15) based on removal/maintaining of the SL. In groups A1, B1 and C1 (without SL), final irrigation was done with 3 mL of 17% ethylenediaminetetraacetic acid (EDTA) (MD-ChelCream, Meta Biomed Co., Ltd., Chungbuk, Korea) for 60 sec, followed by 5 mL of 5.25% NaOCl. In groups A2, B2 and C2 (with SL), the final irrigation protocol was carried out with 5 mL of 5.25% NaOCl for 60 sec. Before obturation the root canals in all groups were completely dried with paper points.

The canals were obturated with cold lateral compaction of gutta-percha and three different sealers; groups A1 and A2 were obturated with AH-26 (Dentsply, Tulsa Dental, Tulsa, OK, USA), groups B1 and B2 with Adseal (Meta Biomed Co., Ltd., Chungbuk, Korea) and groups C1 and C2 with Endofill (Herpo Produtos Dentários Ltda, Petrópolis, RJ, Brazil). Then in all groups the coronal 3 mm of the obturation material was removed and filled with temporary restoration (Sina Dent, Tehran, Iran). 

All teeth were then stored in an incubator at 37^°^C and 100% humidity for 2 days. Then, the samples were coated with two layers of nail varnish except for a 2-mm area around the apical foramen. After 1 h of drying, all the specimens were immersed in India ink (Pelikan, Hannover, Germany) for 72 h at 37^°^C. Then, the teeth were washed in water and the nail varnish was removed with a scalpel blade. These samples were longitudinally sectioned and observed under a stereomicroscope (Nikon, Nikon Corporation, Tokyo, Japan) with 20× magnification for assessing and measuring the linear dye penetration. 

Apical leakage was defined as the distance from the anatomical apex to the deepest extent of vertical dye penetration in a coronal direction. Dye penetration was scored as follows: *score 0*-no dye penetration; *score 1*-dye penetration <0.5 mm; *score 2*-dye penetration from 0.5 to1 mm; *score 3*-dye penetration from 1 to 2 mm; *score 4*-dye penetration ≥2 mm and *score 5*-total dye penetration or through-and-through [18]. In the negative control group the same preparation and filling method was used but the whole root surfaces were coated with two layers of nail varnish. The positive control group included the prepared canals that were filled without sealer. The amount of dye penetration was measured by two independent observers who were blind to the experiment. For each specimen, an average of three readings was recorded. Data were analyzed with descriptive statistical methods and two-way ANOVA using SPSS software (SPSS version 13.0, SPSS, Chicago, IL, USA). The level of significance was set at 0.05.

**Results**

The mean values of dye penetration in AH-26, Adseal and Endofill (A, B and C) groups were 2.53±0.89, 2.76 ±0.93 and 3.03±0.76 mm, respectively. The differences in dye penetration between the three groups were not statistically significant (*P*=0.09). The mean values of dye penetration in the samples obturated with resin sealers (AH-26 and Adseal) was not statistically significant with/without SL (*P*=0.63 for AH-26 and *P*=0.63 for Adseal). The means of dye penetration in Endofill samples with/without SL was not statistically significant, either (*P*=0.63) (Table 1). Almost all the samples gained score 4 of the dye penetration.

**Figure 1:**
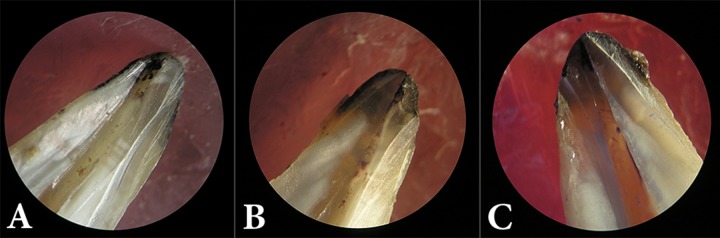
Stereomicroscope photos showing dye penetration; *A)* AH-26, *B)* Adseal and *C)* Endofill (20× magnification)

## Discussion

This study evaluated the microleakage of obturated root canals in presence/absence of SL focusing on the importance of apical seal of the root canal system. The results of this study showed no statistically significant differences in the mean values of dye penetration between the three tested root canal sealers naming AH-26, Adseal and Endofill. Differences in the means of dye penetration between groups with and without the SL were not significant, either. All samples in the positive control group showed apical microleakage, demonstrating the importance of using a sealing material in the canals. None of the samples in negative control group showed leakage. 

The sealing ability needs to be tested for every root canal filling material or technique [19-21]. Several methods have been employed to evaluate the sealing ability of root canal filling materials, including dye penetration, bacterial penetration, radioisotopes, fluorometric assay, electrochemical and scanning electron microscopy (SEM), root clearing, fluid filtration and glucose penetration method [7, 10, 17, 21-23]. 

The dye penetration methodology is based on immersion of sample in various types of dyes (eosin, methylene blue, black India ink, Procion brilliant blue, *etc.*). It was first reported by Grossman and still is being widely used, mainly because it is easy to perform [9, 11, 12, 24-26]. The phenomenon of capillarity is very important in this passive method, as the tooth apex is submerged in the dye that penetrates through any space between the canal walls and the canal filling material [26]. Then the teeth are sectioned longitudinally or transversely or cleared and the linear penetration of dye is recorded [16, 27, 28]. The efficacy of dye penetration method is proved [29]; therefore, we used this method to measure apical microleakage of root canals. 

De Almeida *et al*. [30] evaluated the sealing ability of three endodontic sealers [ZOE-based, glass ionomer-based and resin-based (AH-Plus)] by dye penetration method and all tested sealers showed leakage. The leakage between ZOE- and glass ionomer-based sealers was not significantly different but AH-Plus showed much better sealing ability. However, in the present study resin-based endodontic sealers (AH-26 and Adseal) did not show better seal than ZOE-based sealer (Endofill). This can be attributed to the fact that here the samples were stored at 37^°^C and 100% humidity similar to human body. Also it may be due to the different components of AH-Plus compared to other resin-based sealers like AH-26 and Adseal. 

The SL is created after root canal preparation and consists of organic and non-organic components (vital or necrotic pulp tissue, bacterial components and irritants), which occlude the dentinal tubules [31]. In this study 17% EDTA was used accompanied by 5.25% NaOCl to remove the SL because organic and non-organic particles of the SL are soluble in NaOCl and acids, respectively [32]. It is showed that the presence of SL on canal walls prevents penetration of filling materials into dentinal tubules, and obturation of the canal after SL removal leads to less apical leakage [5, 33]. 

Using the fluid filtration method, Timpawat *et al.* [34] showed that microleakage in samples obturated with thermoplasticized gutta-percha and glass ionomer cement as sealer was more after SL removal. Asnaashari *et al.* [24] showed that dye penetration was less in lased samples because of better removal of the SL by laser. Also khedmat *et al.* [25] demonstrated that the removal of SL can significantly improve the apical sealing ability of AH-26 using electrochemical method. However, DU and Zhu [35] evaluated the apical microleakage of AH-Plus, RoekoSeal, and Calcibiotic Root Canal Sealer (CRCS) with and without the SL; according to their results removal of the SL did not significantly decrease microleakage which is consistent with the results of the present study. These controversial results might be attributed to different methodologies and materials used for evaluation of microleakage [36]. 

In the current study, removal of the SL in three test groups was not significantly effective in preventing apical microleakage. Further investigations are recommended.

## Conclusion

Based on the results of the present study, no significant differences were not observed in the means of dye penetration between AH-26, Adseal and Endofill; AH-26 was superior to other sealers in preventing apical microleakage. Also presence of smear layer did not significantly affect the sealing ability of tested sealers. 
